# Test–Retest Reliability of Self-Reported Sexual Behavior History in Urbanized Nigerian Women

**DOI:** 10.3389/fpubh.2017.00172

**Published:** 2017-07-17

**Authors:** Eileen O. Dareng, Sally N. Adebamowo, Olabimpe R. Eseyin, Michael K. Odutola, Paul P. Pharoah, Clement A. Adebamowo

**Affiliations:** ^1^Department of Public Health and Primary Care, University of Cambridge, Cambridge, United Kingdom; ^2^Institute of Human Virology Nigeria, Abuja, Nigeria; ^3^Division of Cancer Epidemiology, Department of Epidemiology and Public Health, University of Maryland School of Medicine, Baltimore, MD, United States; ^4^University of Maryland Marlene and Stewart Greenebaum Comprehensive Cancer Center, University of Maryland School of Medicine, Baltimore, MD, United States; ^5^Department of Epidemiology, Harvard T.H Chan School of Public Health, Boston, MA, United States; ^6^Institute of Human Virology, University of Maryland School of Medicine, Baltimore, MD, United States

**Keywords:** test–retest reliability, sexual behavior history, interviewer administered questionnaires, self reported behavior, short term variability

## Abstract

**Background:**

Studies assessing risk of sexual behavior and disease are often plagued by questions about the reliability of self-reported sexual behavior. In this study, we evaluated the reliability of self-reported sexual history among urbanized women in a prospective study of cervical HPV infections in Nigeria.

**Methods:**

We examined test–retest reliability of sexual practices using questionnaires administered at study entry and at follow-up visits. We used the root mean squared approach to calculate within-person coefficient of variation (CV_w_) and calculated the intra-class correlation coefficient (ICC) using two way, mixed effects models for continuous variables and $\left(\hat{\text{κ}}\right)$ statistics for discrete variables. To evaluate the potential predictors of reliability, we used linear regression and log binomial regression models for the continuous and categorical variables, respectively.

**Results:**

We found that self-reported sexual history was generally reliable, with overall ICC ranging from 0.7 to 0.9; however, the reliability varied by nature of sexual behavior evaluated. Frequency reports of non-vaginal sex (agreement = 63.9%, 95% CI: 47.5–77.6%) were more reliable than those of vaginal sex (agreement = 59.1%, 95% CI: 55.2–62.8%). Reports of time-invariant behaviors were also more reliable than frequency reports. The CV_w_ for age at sexual debut was 10.7 (95% CI: 10.6–10.7) compared with the CV_w_ for lifetime number of vaginal sex partners, which was 35.2 (95% CI: 35.1–35.3). The test–retest interval was an important predictor of reliability of responses, with longer intervals resulting in increased inconsistency (average change in unreliability for each 1 month increase = 0.04, 95% CI = 0.07–0.38, *p* = 0.005).

**Conclusion:**

Our findings suggest that overall, the self-reported sexual history among urbanized Nigeran women is reliable.

## Introduction

Information about sexual behavior and sexual health is often collected in epidemiologic studies. These include research on risk factors for acquisition and spread of communicable diseases such as sexually transmitted diseases including HIV/AIDS and on non-communicable diseases such as cancers. Sexual history is relevant in diseases where it does not have direct etiological relationship but where it may provide insights into overall well-being and quality of life, or where disease may negatively affect the sexual domain. Examples include chronic illnesses such as stroke and diabetes, whose progression, treatment, or resolution may affect sexual function and quality of life.

In most epidemiologic studies, the history of sexual practices and sexual hygiene are elicited through self-reports but the validity of such data has been repeatedly questioned (1–3). In the absence of precise biomarkers that can serve as gold standards to evaluate the accuracy of self-reports, several studies have been done to evaluate methods of testing the reliability. The most popular method is the use of test–retest correlation of responses to questionnaires while another method uses the presence of biomarkers of vaginal exposure to semen such as the presence of sperm, prostate-specific antigen, or Y chromosome in vaginal fluids (4–8). These latter methods are more relevant for evaluation of recent unprotected sexual intercourse in women and may not be relevant in most epidemiological studies where long-term exposure and variety of exposures are of interest (9). Other methods that have been used include correlation of partner reports of sexual behavior and the use of sexual diaries (2, 9). Partner’s reports of sexual behavior is not ideal because it may be influenced by the nature of the relationship between partners and raises problems of confidentiality in reporting on the behavior of another. The use of sexual diaries in large, population-based studies may not be practicable because of the burden on research participants, which may lead to high attrition rates, non-compliance, recall bias, and participants’ reactivity (2, 9–11).

Studies that used test–retest correlations for measuring reliability of sexual history among diverse populations in the United States have yielded intraclass correlation coefficients (ICC) ranging from 0.3 to 0.9 for reports of lifetime number of sexual partners (9, 12–15). Most studies in low- and middle-income countries (LMICs) that have evaluated the reliability of self-reported sexual history have been restricted to either the young (15–24 years old) or to practitioners of high-risk sexual behavior (16, 17). One possible reason for high prevalence of these types of research in LMIC is that sexual behavior is commonly used as indicators for monitoring the HIV/AIDS epidemic by the Joint United Nations Program on HIV/AIDS (UNAIDS) (18). As self-report of sexual behavior may be subject to self-presentation and social desirability bias, which may differ by age, sex, and population characteristics that reflect acceptable norms and cultural attitudes toward talking about sex in any given society, it is important to evaluate reliability in the context of conducting epidemiological research in resource limited settings (6).

In this study, we examined a 14-item questionnaire used to collect sexual behavior history from urbanized Nigerian women to determine its reliability and that of similar instruments used for self report of sexual behavior in epidemiological research.

## Materials and Methods

### Study Population

Between August 2012 and December 2013, we recruited women from our cervical cancer screening clinics in Abuja, Nigeria, into a prospective study of the host and viral factors associated with persistent hrHPV infection in Nigeria. We enrolled women who were at least 18 years old and had engaged in vaginal sexual intercourse. We excluded women who had a total hysterectomy, were pregnant, or unable to provide an informed consent. At enrollment, we used interviewer administered questionnaires to collect data on sociodemographic characteristics, lifestyle risk factors, reproductive and sexual behavior histories. Trained nurses performed gynecologic examinations on all participants, collected biological samples for HPV detection, and examined the cervix for premalignant lesions through visual inspection with acetic acid/Lugol’s reagent (VIA/VILI). We treated all women diagnosed with premalignant cervical lesions with thermocoagulation if the lesions met specific criteria: complete visualization of the lesions, lesions covering less than 75% of the transformation zone, lesions amenable to complete coverage by the tip of the cryoprobe and lesions not suspicious of cancer (19). All participants were scheduled for a follow-up visit after 6 months. At the follow-up visit, the same nurses who administered the baseline questionnaires readministered the questionnaires to all returning participants. At the baseline visit, participants were not informed that they would be asked the same questions at follow-up. All nurses were trained to administer the questionnaires either in English or local languages in cases where participants could not speak English. All questionnaires were completed prior to biological sample collection.

### Main Outcome Measures

We adapted protocols for sexual behavior history from the Phenx toolkit version 5.0 February 24, 2012 and developed a 14-item questionnaire. We piloted the questionnaires among 50 women of reproductive age with similar characteristics as our study population. Details of these items and coding of responses are shown in Table 1. Six items were coded as continuous variables while eight items were coded as categorical. To distinguish between actual behavior change and test–retest reliability, we asked all participants to report changes in sexual behavior in the period between the first and second questionnaires, and adjusted our analysis to account for any reported changes.

**Table 1:** Sexual behavior history questionnaire items.

Variables	Question format	Responses permitted
**Continuous variables**		
Age at sexual initiation	How old were you the first time you had any type of sex?	Open ended
Lifetime number of partners	How many partners have you had any type of sex with in total, over the years?	Open ended
Age at oral sex debut	How old were you the first time you had oral sex?	Open ended
Lifetime number of oral sex partners	How many partners have you had oral sex with in total, over the years?	Open ended
Age at anal sex debut	How old were you the first time you had anal sex?	Open ended
Lifetime number of anal sex partners	How many partners have you had anal sex with in total, over the years?	Open ended
**Categorical variables**		
Sexual orientation	What type of sexual relations do you usually have?	With men only With women only With men and women I don’t want to talk about it
Type of sex at sexual debut	What type of sex did you have the first time you had sex?	Oral Anal Vaginal Any combination of the above
Ever practiced oral sex	Have you ever had oral sex?	Yes No
Type of oral sex usually engaged in	Which type of oral sex do you usually engage in?	Fellatio (mouth to male genitals) Cunnilingus (mouth to female genitals) Anallingus (mouth to anus) Any combination of the above
Ever practiced anal sex	Have you ever had anal sex?	Yes No
Frequency of vaginal sex	How frequently do you engage in vaginal sex	Daily More than once in a week but not daily Once, or twice, or thrice a month Once or twice in last 3 months Once in last 6 months Once a year
Frequency of oral sex	How frequently do you engage in oral sex	Daily More than once in a week but not daily Once, or twice, or thrice a month Once or twice in last 3 months Once in last 6 months Once a year
Frequency of anal sex	How frequently do you engage in anal sex	Daily More than once in a week but not daily Once, or twice, or thrice a month Once or twice in last 3 months Once in last 6 months Once a year

### Statistical Analysis

For categorical variables, we estimated kappa coefficient $\left(\hat{\text{κ}}\right)$ to determine agreement beyond what would be expected by chance. We estimated 95% confidence intervals (95% CI) for $\hat{\text{κ}}$ using bootstrap methods with bias-corrected estimation as some of the variables such as type of sex at sexual debut, and types and frequency of practice of different types of sex had more than two categories (20–22). We compared the $\hat{\text{κ}}$ statistics for HIV-negative and HIV-positive women using the *z* statistic (23). We used the Landis and Koch benchmarks to interpret kappa values: <0.00 (poor), 0.00–0.20 (slight), 0.21–0.40 (fair), 0.41–0.60 (moderate), 0.61–0.80 (substantial), 0.81–1.00 (almost perfect) (24).

For continuous variables, we calculated indices of absolute and relative test–retest reliability. For absolute reliability, the degree to which repeated responses varied for individuals, we used within person coefficients of variation (CV_w_), Bland and Altman’s limit of agreement, paired *t*-tests, differences in responses at study entry and retest. For relative reliability, the degree to which individuals maintain their position in the group, we used ICCs two-way mixed effects model. We chose to use the two-way mixed effects ICC model because the same set of research assistants administered the same questionnaires to all participants at study entry and retest. Therefore, the research assistants and questionnaires were considered to be fixed effects while the random effects were participants and possibly the interactions between participants and the research assistants. We used the guidelines suggested by Cicchetti to interpret the correlation coefficients, with values below 0.40 interpreted as poor; values of 0.40–0.59 as fair; values of 0.60–0.74 as good, and values of 0.75–1.00 as excellent (25).

To investigate the association between potential correlates and test–retest reliability, we used two different types of regression models; log binomial regression models for sexual behavior responses collected as categorical variables; and linear regression models for sexual behavior response collected as continuous variables (Table 1). We evaluated age-adjusted models of the outcome and the potential predictors such as interval between test administration, marital status, level of education, self-perception of general health and HIV status, and others identified from the literature. We identified predictors with *p*-values less than 0.20 in the age-adjusted models and included them in multivariable regression models (9, 26).

We used principal component analysis to create a summary measure of reliability for the continuous variables. Using the eigenvalue cutoff of 1, the scree plot, and interpretability of factors, we retained one factor, which explained a cumulative variance of 53%. We predicted scores for test–retest reliability using the factor loadings for the retained factor, such that participants with high scores may be considered to have higher levels of inconsistency in their responses compared with participants with low scores. We used the summary measure in linear regression models testing for test–retest reliability for each participant (Table 1).

For the categorical variables (Table 1), we created a summary variable such that participants who had any disagreement in the categorical variables at test and retest had a score of one and participants who were consistent in their test–retest responses had a score of 0. Next, we used this summary measure in log binomial models to evaluate the association between potential correlates and reliability of responses provided for sexual behavior questions collected as categorical variables (Table 1).

We considered a *p*-value <0.05 as significant. Formal adjustments for multiplicity were not considered appropriate as inferences for itemized questionnaire items were not based on significance of individual endpoints. In regression models, where inferences were based on significance of the endpoint, we used summary variables as endpoints. All statistical analyses were conducted using Stata version 13 (Stata Corp, College Station, TX, USA).

## Results

### Study Characteristics

Of the 725 participants included in this study, 346 (48%) were HIV positive, 354 (49%) were HIV negative, and the HIV status of 25 (3%) participants was unknown. The latter were excluded from regression models and comparisons of reliability between HIV-positive and HIV-negative participants. The mean (SD) age of participants was 38.5 (7.8) years and mean (SD) interval between questionnaire administrations was 8.6 (4.0) months (Table 2). Most of the participants were married (67%) and had more than 6 years of formal education (88%). The prevalence of oral and anal sex among the participants at study entry was 16 and 2%, respectively.

**Table 2:** Characteristics of study participants at enrollment.

	Total^a^	HIV positive	HIV negative	*p*-Value
	*N* = 725	*N* = 346	*N* = 354	

	Mean (SD)			
Age (years)	38.5 (7.8)	37.5 (7.4)	39.6 (7.9)	<0.001
Interval between visits (months)	8.6 (4.0)	9.0 (4.6)	8.1 (3.3)	<0.001
		***N* (%)**		
Current marital status				
Married	480 (66.9)	183 (53.0)	284 (80.4)	<0.001
Unmarried	238 (33.1)	162 (47.0)	69 (19.6)	
Education				
6 years or less	86 (12.0)	44 (12.8)	38 (10.8)	0.42
More than 6 years	632 (88.0)	301 (87.2)	315 (89.2)	
Alcohol consumption in past 3 months	96 (13.6)	50 (14.7)	43 (12.4)	0.38
Presence of a chronic condition^b^	152 (21.3)	91 (26.2)	56 (16.3)	<0.001
Ever practiced oral sex^c^	127 (17.7)	59 (17.1)	63 (17.8)	0.66
Type of oral sex practiced^c^				
Fellatio	33 (4.6)	15 (4.3)	18 (5.1)	
Cunnilingus	22 (3.0)	13 (3.8)	8 (2.3)	0.43
Analingus	2 (0.3)	2 (0.6)	0 (0.0)	
Combination	65 (9.0)	27 (7.8)	34 (9.6)	
Ever practiced anal sex^c^	15 (2.2)	7 (2.1)	8 (2.4)	0.83
Type of anal sex practiced^c^				
Receptive	5 (0.7)	3 (0.9)	2 (0.6)	
Insertive (sex toys)	2 (0.3)	0 (0.0)	2 (0.6)	0.61
Both	1 (0.1)	0 (0.0)	1 (0.3)	
Sexual orientation^c^				
Men only	716 (99.9)	352 (99.7)	344 (100.0)	1.00
Men and women	1 (0.1)	1 (0.3)	0 (0.0)	

*^a^Includes participants whose HIV status was unknown*.

*^b^Chronic conditions listed were hypertension, diabetes, peptic ulcer disease, Hepatitis B*.

*^c^At study entry*.

### Indices of Absolute Reliability

**Figure 1:**
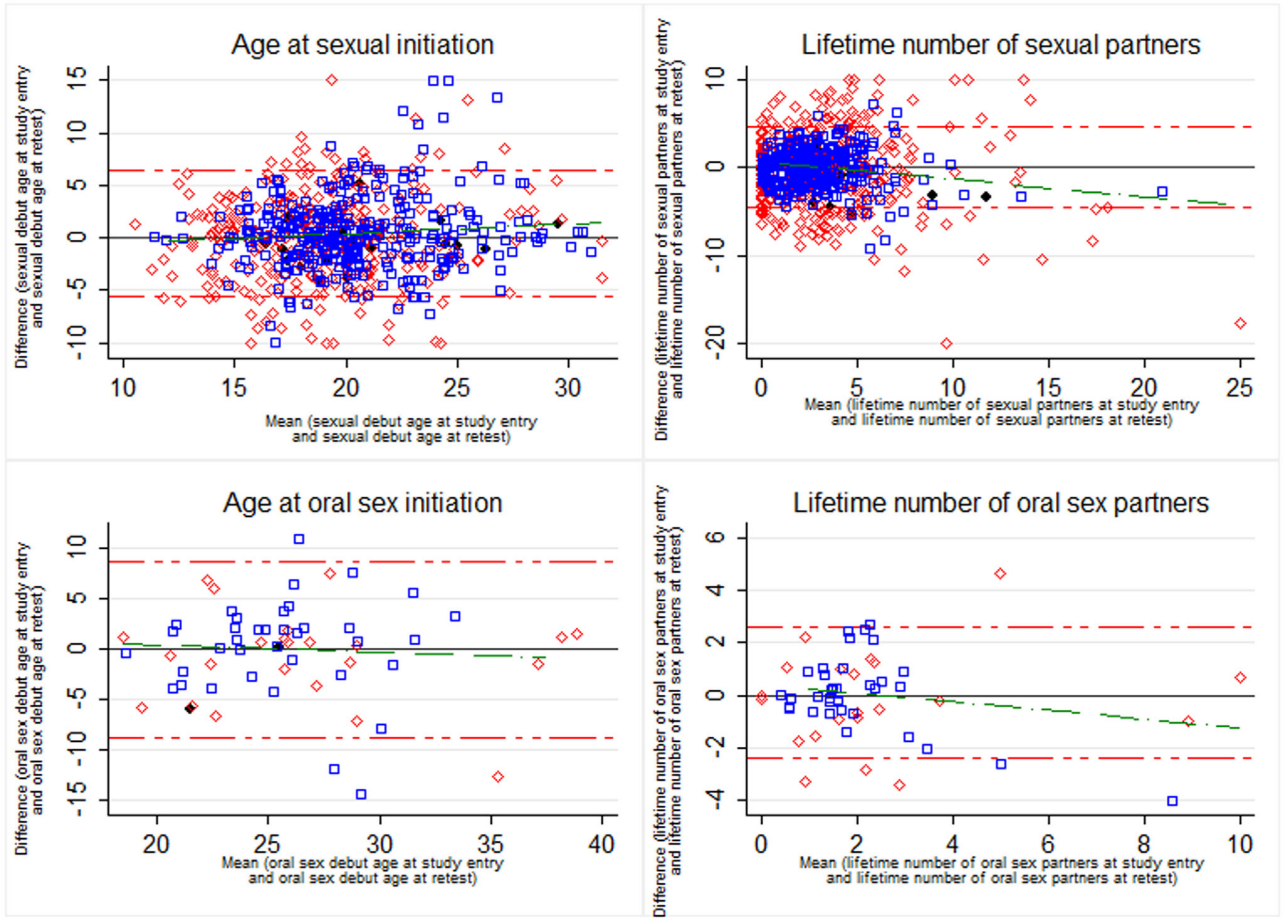
Bland and Altman plots of responses provided at study entry and retest for age at sexual initiation, age at oral sex initiation, lifetime number of sexual partners, and lifetime number of oral sex partners. 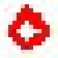 represent HIV-negative women, 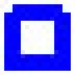 represent HIV-positive women, and 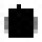 represents women with unknown HIV status. The red etched lines represent the 95% agreement limits (1.96 × SD of the differences). The green etched line represents a regression line fitting the paired differences to the pairwise means.

The mean of the difference (SD) in responses provided at study entry and at retest for all but one of the continuous variable was close to 0 [age at sexual debut, 0.4 (3.1); lifetime number of partners, 0.0 (2.3); age at oral sex debut, −0.1 (4.5); lifetime number of oral sex partners 0.1 (1.3); age at anal sex debut −1.0 (6.2)] (Table 3). Except for age at oral and anal sex debut, the responses provided at retest were generally lower than the responses provided at baseline as shown by the positive direction of the mean of the difference between the responses (Table 3). The 95% limits of agreement for the mean of the differences between responses at study entry and retest are shown in the Bland and Altman plots (Figure 1). The plots show that with increasing number of sexual partners reported, the less reliable the responses were. Comparing HIV-negative women to HIV-positive women in univariate analyses, there were no significant differences for the sexual history measures collected as continuous variables: age at sexual initiation (*p* 0.25), lifetime number of partners (*p* 0.86), age at oral sex debut (*p* 0.61), lifetime number of sexual partners (*p* 0.76), and age at anal sex debut (*p* 0.76).

**Table 3:** Absolute and relative indices for test–retest reliability of sexual behavior history.

	Indices of absolute test–retest reliability							Index of relative test retest reliability	
Variable	Enrollment mean (SD)	Retest mean (SD)	*p*-Value (paired *t*)	Mean difference (SD)^a^	95% limits of agreement^b^	*p*-Value^c^	CV_w_ (95% CI)^d^	ICC (95% CI)	*p*-Value^e^
**General sex practice**									
Age at sexual debut (*N* = 663)	20.1 (3.8)	19.8 (3.6)	0.001	0.4 (3.1)	−5.6 to 6.4		10.7 (10.6–10.7)	0.8 (0.8–0.8)	
HIV positive (*N* = 315)	19.4 (3.6)	19.2 (3.4)	0.13	0.3 (3.0)	−5.6 to 6.2	0.25	10.9 (10.8–10.9)	0.8 (0.7–0.8)	0.51
HIV negative (*N* = 330)	20.8 (3.9)	20.3 (3.6)	0.002	0.5 (3.1)	−5.6 to 6.7		10.6 (10.6–10.7)	0.8 (0.8–0.8)	
Lifetime number of partners (*N* = 682)	3.3 (2.6)	3.3 (3.0)	0.63	0.0 (2.3)	−4.5 to 4.6		35.2 (35.1–35.3)	0.8 (0.8–0.8)	
HIV positive (*N* = 323)	3.8 (2.8)	3.7 (3.6)	0.64	0.1 (2.9)	−5.7 to 5.9	0.86	38.8 (38.6–39.0)	0.8 (0.7–0.8)	<0.001
HIV negative (*N* = 339)	2.8 (2.2)	2.8 (2.2)	0.61	0.0 (1.6)	−3.0 to 3.1		31.7 (30.0–33.4)	0.9 (0.8–0.9)	
**Oral sex practice**									
Age at oral sex debut (*N* = 62)	26.3 (5.5)	25.8 (5.2)	0.93	−0.1 (4.5)	−8.8 to 8.7		11.5 (11.5–11.6)	0.7 (0.5–0.8)	
HIV positive (*N* = 23)	25.9 (5.6)	26.0 (6.2)	0.67	−0.3 (3.8)	−7.9 to 7.2	0.61	9.9 (9.7–10.0)	0.9 (0.7–1.0)	
HIV negative (*N* = 37)	26.7 (5.5)	25.6 (4.4)	0.74	0.3 (4.9)	−9.3 to 9.8		12.3 (12.1–12.5)	0.4 (0.1–0.7)	0.001
Lifetime number of oral sex partners (*N* = 57)	1.8 (1.5)	1.8 (1.7)	0.03	0.1 (1.3)	−2.4 to 2.6		34.1 (33.9–34.4)	0.9 (0.8–0.9)	
HIV positive (*N* = 36)	2.0 (2.0)	2.0 (2.0)	1.00	0.0 (1.4)	−2.7 to 2.7	0.76	34.3 (33.5–35.1)	0.9 (0.8–1.0)	0.05
HIV negative (*N* = 21)	1.7 (1.0)	1.6 (1.5)	0.60	0.1 (1.3)	−2.4 to 2.6		33.6 (33.1–34.0)	0.8 (0.6–0.9)	
**Anal sex practice**									
Age at anal sex debut (*N* = 5)	24.0 (8.2)	27.1 (10.4)	0.73	−1.0 (6.2)	−5.4 to 3.4		–	0.9 (0.2–1.0)	
HIV positive (*N* = 1)	25.0 (0.0)	23.0 (0.0)	–	2.0 (−)	–	0.76	–	–	–
HIV negative (*N* = 4)	24.6 (10.6)	27.4 (10.5)	0.65	−1.8 (7.0)	−15.4 to 11.9		–	0.9 (−0.2 to 1.0)	

*CI, confidence interval; CV_w_, within-person coefficient of variation; ICC, intra-class correlation coefficient; ρ, Spearman rank’s correlation coefficient*.

*^a^Mean difference between response at enrollment and response at follow-up*.

*^b^Mean ± 1.96 × SD*.

*^c^Student’s t-test was used to compare mean difference for HIV positive and HIV negative participants*.

*^d^The CV_w_ was calculated using the root mean squared approach and confidence intervals obtained by bootstrap methods*.

*^e^Fishers *z* transformation was used to compare correlation coefficients of HIV-positive and HIV-negative women (27)*.

The intraindividual variability were lower for time-invariant measures (age at sexual debut CV_w_ = 10.7 and age at oral sex debut CV_w_ = 11.5) compared with frequency measures (lifetime number of partners CV_w_ = 35.2, and lifetime number of oral sex partners CV_w_ = 34.1) (Table 3).

### Indices of Relative Reliability

As shown in Table 3, the ICC for age at sexual debut (0.8), total lifetime number of partners (0.8), age at oral sex debut (0.7), and total lifetime number of oral sex partners (0.9) for the total study population was excellent.

HIV-negative women had a ICC than HIV-positive women for lifetime number of partners (0.9 vs 0.8, *p* < 0.001). Conversely, HIV-negative women had a lower ICC than HIV-positive women for age at oral sex debut (0.4 vs 0.9, *p* 0.001).

### Agreement for Categorical Variables

There was a high level of agreement between responses at study entry and responses at retest for sexual orientation (98.8%), type of sex at sexual debut (97.8%), ever practiced oral sex (85.4%), ever practiced anal sex (98.2%) (Table 4). However, agreement for frequency of sexual activity was relatively lower ranging from 59.1% for frequency of vaginal sex to 63.9% for oral sex. Despite the high levels of agreement, $\hat{\text{κ}}$ statistics were slight to moderate. Generally, HIV-negative individuals had higher $\hat{\text{κ}}$ statistics than HIV-positive individuals.

**Table 4:** Test–retest reliability of self-reported sexual history using categorical variables.

Variable	Agreement (%) (95% CI)	$\hat{\text{κ}}$ (95% CI)	*p*-Value
**General sex practice**			
Sexual orientation (*N* = 689)	98.8 (97.7–99.5)	0.2 (0.0–0.6)	
HIV positive (*N* = 330)	99.4 (97.7–100.0)	0.0 (0.0–0.0)	<0.001
HIV negative (*N* = 339)	98.2 (96.1–99.3)	0.3 (0.0–0.7)	
Type of sex at sexual debut (*N* = 669)	97.8 (96.3–98.7)	0.1 (0.0–0.4)	
HIV positive (*N* = 317)	97.5 (95.0–98.8)	0.0 (0.0–0.0)	<0.001
HIV negative (*N* = 333)	98.5 (96.4–99.5)	0.3 (0.0–0.7)	
Frequency of vaginal sex (*N* = 640)	59.1 (55.2–62.8)	0.3 (0.3–0.4)	
HIV positive (*N* = 306)	55.2 (49.6–60.7)	0.3 (0.2–0.3)	0.17
HIV negative (*N* = 315)	62.2 (56.7–67.4)	0.3 (0.2–0.4)	
**Oral sex practice**			
Ever practiced oral sex (*N* = 693)	85.4 (82.0–87.3)	0.5 (0.4–0.6)	
HIV positive (*N* = 332)	83.5 (78.4–86.5)	0.4 (0.2–0.5)	0.01
HIV negative (*N* = 341)	87.3 (83.1–90.3)	0.6 (0.5–0.7)	
Type of oral sex usually engaged in (*N* = 62)	54.8 (42.5–66.0)	0.2 (0.0–0.4)	
HIV positive (*N* = 24)	50.0 (31.4–68.6)	0.2 (0.1–0.5)	0.43
HIV negative (*N* = 36)	55.6 (39.6–70.5)	0.2 (0.1–0.5)	
Frequency of oral sex (*N* = 36)	63.9 (47.5 -77.6)	0.4 (0.2–0.6)	
HIV positive (*N* = 17)	64.7 (41.2–82.8)	0.4 (0.1–0.8)	0.48
HIV negative (*N* = 19)	63.2 (40.9–81.0)	0.4 (0.1–0.8)	
**Anal sex practice**			
Ever practiced anal sex (*N* = 688)	98.0 (96.6–98.8)	0.5 (0.2–0.7)	
HIV positive (*N* = 330)	97.3 (94.8–98.6)	0.2 (0.0–0.6)	<0.001
HIV negative (*N* = 338)	98.5 (96.5–99.5)	0.6 (0.2–0.9)	

### Predictors of Reliability

In Model 1 for continuous variables, we found that a 1-month increase in test–retest interval resulted in an average increase of 0.04 points in inconsistency of responses (95% CI = 0.01–0.06, *p-*value = 0.003) (Table 5). HIV infection was also statistically significantly associated with reliability, with HIV-positive individuals having an average increase of 0.22 points in inconsistency compared to HIV-negative individuals (95% CI = 0.07–0.38, *p-*value = 0.005). In Model 2 for categorical variables, we did not observe any significant relationships.

**Table 5:** Regression models for reliability for continuous and categorical questions.

	Model 1		Model 2	
Variables	Average change in unreliability score (95% CI)	*p*-Value	RR (95% CI)	*p*-Value
Retest interval	0.04 (0.01–0.06)	0.003	1.01 (0.99–1.02)	0.44
HIV status				
HIV negative	Reference		Reference	
HIV positive	0.22 (0.07–0.38)	0.005	1.17 (0.99–1.39)	0.07
Age	0.00 (0.00–0.01)	0.85	0.99 (0.9–1.00)	0.22
Current marital status				
Married	Reference		Reference	
Unmarried	0.04 (−0.12–0.21)	0.62	1.01 (0.86–1.21)	0.92
Perception of general health				
Excellent	Reference		Reference	
Good	0.05 (−0.11–0.22)	0.54	0.95 (0.79–1.14)	0.56
Fair	0.10 (−0.12–0.34)	0.38	0.86 (0.67–1.10)	0.23
Poor	−0.16 (−0.59–0.27)	0.46	1.52 (0.96–2.42)	0.08
Level of education				
6 years of formal education or less	Reference		Reference	
More than 6 years of formal education	0.17 (−0.03–0.38)	0.09	1.19 (0.90–1.59)	0.23

*CI, confidence interval; RR, relative risk*.

*Model 1: multivariable linear regression model. Outcome variable in this model is a summary measure of reliability obtained from principal component analysis of responses to open ended sexual behavior questionnaire items (Table 1)*.

*Model 2: log binomial regression model. Outcome variable in this model is a summary measure such that participants who had any disagreement between responses at study entry and responses at retest for the close ended sexual behavior questionnaire (Table 1) items were coded as 1 and 0 otherwise*.

## Discussion

In this study of test–retest reliability of self-reported sexual behavior using interviewer administered questionnaires, we found that self-report of sexual behaviors was reasonably reliable overall. However, we observed varying levels of reliability based on the nature of sexual behavior reported. The reports on frequency of non-vaginal sexual practices were more reliable than those of vaginal sexual practices. Differences in the patterns of reliabilities for frequency of vaginal and non-vaginal sexual practices may reflect differences in the frequencies of the behaviors. Among heterosexual women, vaginal sexual practices tend to occur more frequently than non-vaginal sexual practices (14). Reports of less frequent behavior are generally more stable, as people tend to use more efficient recall strategies (28, 29). Enumeration recall strategies, where each event is recalled and counted separately are commonly used for infrequent behaviors, especially when these behaviors are associated with particularly distinctive time periods, events, or people. However for frequent behaviors, enumeration may be too difficult or time consuming; therefore, estimation recall strategies where rate-based mental calculations are made without recalling individual events are commonly used (30–32).

We found that reports of time-invariant events (age at sexual debut, ever-practiced oral sex, ever-practiced anal sex) were more reliable than frequency reports (number of partners, frequency of sex). This finding may reflect the different psychological processes that underlie these two types of reports. Time-invariant events may be associated with more vividness and personal salience, especially when accompanied with strong emotions at the time of the encounter, for example, age at sexual initiation or ever practiced anal sex (9). Conversely, frequency reports that asks about number of events may involve less vivid memories especially in people with high levels of sexual networking. This is further complicated by the need for rate-based inferences, which require mental calculations that can be inconsistent (9, 11, 30).

For continuous measures, reliability was also significantly decreased with increasing interval between questionnaire administration after controlling for age, HIV status, marital status, perception of general health, and level of education. One possible explanation for our finding is the possibility that behaviors may change with increasing intervals between tests and, therefore, responses provided at retest may reflect current behavior at the time of test administration rather than an indication of instability. Several research studies have evaluated the relationship between recall periods and reliability. Results from some of these studies showed that shorter recall periods were more reliable than longer recall periods (9, 33). On the other hand, other studies reported no association or increased reliability for longer recall periods for particular behaviors such as lifetime number of sexual partners (15, 30, 34). These varying results may reflect underlying differences in the nature of behaviors evaluated, mode of assessment, and study population as can be observed from the results from our models that evaluate reliability for variables collected as categorical where there were no associations between HIV status, test–retest interval, and reliability. While our results provide the only estimates for women living in an urban community in Nigeria, similar findings have been reported in adolescent populations in South Africa (35). The optimal recall period for studies on sexual risk remains an active area of research (2).

Although the indices for absolute test–retest reliability for lifetime number of partners showed high levels of reliability, responses were less reliable as the self-reported number of partners increased, which is consistent with results from previous studies (10, 29, 30, 36). This may be explained by a combination of several factors, such as different recall strategies and the attitudinal propensity toward casual sex among people with multiple sexual partners compared to people who claim to be abstainees or monogamists (30). Participants who have been sexually inactive or monogamous during the recall period may use enumeration strategies to report 0 or 1, respectively. Whereas participants who have had multiple sexual partners may use rate-based mental calculations, which yield imprecise estimates. The cognitive processes involved in the abstinent or monogamous participant are straightforward and probably result in higher degrees of reliability than in women reporting multiple sexual partners. Additionally, studies show that people with higher numbers of sexual partners display more favorable attitudes toward casual sex, which tend to be less vivid with less psychological involvement than sex in the context of sustained relationships (30). As recall is associated with vividness of events, it is understandable that discrepancies are higher with increasing number of partners (37).

### Strengths of this Study

A notable strength of our study is that we evaluated reliability of individual sexual behaviors, rather than assume that reliability of measures of one sexual behavior confer reliability on other measures of sexual behavior. This has important implications for researchers in making informed decisions about the collection of self reported sexual history.

In estimating sample size for epidemiologic studies, the importance of considering measurement errors of important covariates has been described by several authors (38, 39). One simple approach is to adjust the sample size estimates based on desired level of statistical power and level of precision in the presence of perfect measurements, by the square of the correlation between the true value and the observed covariate value (38). An alternative to sample size adjustments is to incorporate expected levels of measurement error into the data analysis (40). These approaches require that the magnitude of the measurement error for the covariates are known. In the absence of correlation, estimates for true and self-reported sexual behavior history, due to difficulties in determining the true values, our test–retest correlation estimates provide some guidance for sample size adjustments to account for measurement errors in the use of self-reported sexual behavior history in epidemiologic studies. Another strength of our study is that by examining a time-invariant sexual attribute such as age at sexual debut, we were able to evaluate test–retest reliability without the confounding effects of behavior change that may occur during the test interval. For time-variant measures such as lifetime number of sexual partners, we included a question in the retest questionnaire for participants to record number of new partners since the administration of the first test.

Motivation to participate in a research study and topic of research study may be important sources of response bias (41). Participants in reproductive and sexual health research studies may give more thoughtful responses to questions on sexual practices because of altruistic reasons in aiding investigators to arrive at useful answers or they may perceive that their responses may affect their clinical management, leading to better reliability than participants in other types of studies, where sexual behavior may not be perceived as being important. Our study was hospital-based and conducted among adult females in the context of cervical cancer screening; therefore, our participants may have given responses that can be generalized to populations who participate in similar research.

### Limitations

In our study, we used interviewer administered in-person interviews, and this may have led participants to provide more socially desirable responses. We minimized interviewer influences by using well-trained interviewers and by arranging sensitive questions after less sensitive ones so that the participants’ trust would be high by the time sensitive questions were asked. There is some evidence to suggest that participants respond more objectively to self-administered interviews than to interviewer-administered ones, particularly for behaviors that may be considered embarrassing, stigmatizing, or illegal (42). This may be due to increased privacy afforded by self-administered interviews and ability of participants to control the pace of the interview. However, other studies have found no difference in the use of either methods, especially for sexual behavior history that may include complex branch and skip patterns (43). Audio-assisted computer self-administered questionnaires may improve objectivity of self-administered questionnaires, but they require respondents to comprehend questions and provide relevant responses. Their infrastructural demands and literacy requirements may preclude their use in large scale epidemiological studies in LMICs (44, 45).

Although the $\hat{\text{κ}}$ statistic is important in evaluating agreement beyond chance for categorical variables, it is highly dependent on prevalence and marginal totals (46). Thus, low $\hat{\text{κ}}$ values will be obtained despite high percent agreement when prevalence of traits is low, as observed with prevalence of anal sex practice (2.2%), and also when marginal totals are highly asymmetric, as was observed with sexual orientation, and type of sex practiced at sexual debut in this study population.

Given the prevalence of oral and anal sex in this study population, our sample size may have limited power in detecting small differences between responses provided at study entry and retest for questionnaire items on oral and anal sex.

## Conclusion

Our study provides valuable insight on the reliability of sexual behavior history data for studies conducted in developing countries and shows that the overall test–retest reliability of sexual behavioral history among urbanized adult women in Nigeria is high. Relative indices of reliability were generally high and within person variability was higher for frequency measures compared to time-invariant measures. This implies that with well-trained interviewers and carefully formatted questionnaire items, researchers can utilize self-reported sexual history data in epidemiological studies in LMICs.

## Ethics Statement

All participants gave written informed consent in accordance with the Declaration of Helsinki. The protocol was approved by the National Health Research Ethics Committee of Nigeria and the University of Maryland Institutional Review Board.

## Author Contributions

ED, SA, OE, and MO contributed to the acquisition, analysis, interpretation of data, and drafting of the manuscript. PP contributed to the interpretation of data and revising it critically for important intellectual content. CA contributed to the conception, design, interpretation of data, critical revision of the manuscript for intellectual content, and obtained funds for the study. All authors approved the final version of this paper.

## Conflict of Interest Statement

The authors declare that the research was conducted in the absence of any commercial or financial relationships that could be construed as a potential conflict of interest.
